# Cognitive Enhancing and Neuroprotective Effect of the Embryo of the *Nelumbo nucifera* Seed

**DOI:** 10.1155/2014/869831

**Published:** 2014-12-24

**Authors:** Eun Sil Kim, Jin Bae Weon, Bo-Ra Yun, Jiwoo Lee, Min Rye Eom, Kyoung-Hee Oh, Choong Je Ma

**Affiliations:** ^1^Biological and Genetic Resources Utilization Division, National Institute of Biological Resources, Hwangyeong-ro 42, Seo-gu, Incheon 404-708, Republic of Korea; ^2^Department of Medical Biomaterials Engineering, College of Biomedical Science, Kangwon National University, Hyoja-2 Dong, Chuncheon 200-701, Republic of Korea; ^3^Research Institute of Biotechnology, Kangwon National University, Chuncheon 200-701, Republic of Korea

## Abstract

The aim of the present study was to evaluate the effect of ENS on cognitive impairment induced by scopolamine and its potential neuroprotective effect against glutamate-induced cytotoxicity in HT22 cell and to investigate the underlying mechanisms. ENS (3, 10, 30, and 100 mg/kg), scopolamine (1 mg/kg), and donepezil (1 mg/kg) were administered to mice during a test period. Scopolamine impaired memory and learning in a water maze test and a passive avoidance test. The neuroprotective effect of ENS (10 and 100 *μ*g/mL) was investigated on glutamate-induced cell death in HT22 cells by MTT assay. We investigated acetylcholinesterase inhibition in hippocampus and antioxidant activity, ROS levels, and Ca^2+^ influx in HT22 cells to elucidate the potential mechanisms of ENS. We found that ENS significantly ameliorated scopolamine-induced memory impairment and inhibited AChE activity in hippocampus. *In vitro*, ENS showed potent neuroprotective effects against glutamate-induced neurotoxicity in the HT22 cell. In addition, ENS induced a decrease in ROS production and intercellular Ca^2+^ accumulation and showed DPPH radical and H_2_O_2_ scavenging activity. In conclusion, ENS showed both a memory improving effect and a neuroprotective effect. Our results indicate that ENS may be of use in the treatment and prevention of neurodegenerative disorders.

## 1. Introduction

Alzheimer's disease (AD) is neurodegenerative disorder characterized by deficits of memory as well as other cognitive impairments [1]. The defined pathogenesis of AD is amyloid plaque deposits (A*β*), neurofibrillary tangles (NFT), and neuronal cell death [2].

*Nelumbo nucifera* Gaertner, an aquatic plant of the Nelumbonaceae family, is an Asiatic medicine known as “Sacred Lotus.” Almost all parts of* Nelumbo nucifera*, including the leaves, flowers, and rhizomes, are used as food and traditional medicine in Korea, China, and Japan. The embryo of the* Nelumbo nucifera* seed (ENS) is traditionally used to alleviate fever and arrest bleeding [3]. ENS is composed of bisbenzylisoquinoline alkaloids, benzylisoquinoline alkaloids, aporphine, and proaporphine alkaloids [4]. Neferine, a major bisbenzylisoquinoline alkaloid, is the active compound in ENS [5–7]. Previous studies have reported that ENS and neferine show sedative and anxiolytic effects in mice [8].

Various parts of* Nelumbo nucifera* have been reported to exhibit effects associated with Alzheimer's disease. For example,* Nelumbo nucifera* seed protects the mouse embryonic fibroblast cells by inhibiting H_2_O_2_-induced cytotoxicity, and* Nelumbo nucifera* semen improves scopolamine-induced dementia by inhibiting acetylcholinesterase (AChE) activity [9, 10].* Nelumbo nucifera* rhizome also improves memory function by enhancing neurogenesis in the dentate gyrus of the rat hippocampus [11, 12], and procyanidins isolated from the* Nelumbo nucifera* seedpod ameliorate scopolamine-induced memory impairment by inhibiting AChE activity [13].

Based on the findings from various parts of* Nelumbo nucifera*, it might be expected that ENS may also have an improving effect on memory. However, to date there are no reports that ENS has a cognitive-enhancing or neuroprotective effect. The aim of this study was to investigate the cognitive-enhancing effect of ENS, using the Morris water maze test and the passive avoidance test. These tests are widely used to investigate spatial memory and learning function. Scopolamine, a nonselective muscarinic cholinergic receptor antagonist, induces memory impairment and is a valuable* in vivo* model of AD [14]. We therefore evaluated the effect of ENS on scopolamine-induced amnesia in mice, using the Morris water maze test and passive avoidance test and in addition, we evaluated the neuroprotective effect of ENS against glutamate-induced cell death in mouse hippocampal HT22 cells.

## 2. Materials and Methods

### 2.1. Chemical Material

Scopolamine (#S0929), trolox (#238813), ascorbic acid (#A5960), butylated hydroxyanisole (BHA) (#B1253), donepezil (#1224981), 2′7′-dichlorofluorescein diacetate (DCF-DA) (#35845), and Fura-2AM (#F0888) were supplied by Sigma Aldrich Co. Ltd. (USA). Dulbecco's modified Eagle's medium (DMEM) (#D5648) and fetal bovine serum (FBS) (#10437028) were supplied by Gibco BRL Co. (U.S.A).

### 2.2. Plant Material and Sample Preparation

The embryo of the* Nelumbo nucifera* seed was obtained from Wildlife Genetic Resources Center at National Institute of Biological Resources (NIBR). The embryo of the* Nelumbo nucifera* seed sample was authenticated by Dr. Young Bae Seo (a Professor of the College of Oriental Medicine, Daejeon University, Daejeon, Korea) and has been deposited at the Kangwon National University (Chuncheon, Korea) as a voucher specimen (#CJ151 M).

The embryo of the* Nelumbo nucifera* seeds (15.75 g) was extracted with 80% methanol by ultrasonication-assisted extraction at room temperature 3 times and then the methanol extract was evaporated. Dried extract was obtained by freeze-dry.

### 2.3. Animals

3-week-old male ICR mice, weighting 25–30 g, were used in this study. Mice were housed seven per cage and were maintained in temperature 20 ± 3°C under a 12/12 h light/dark cycle and adapted for 1 week before testing began. Commercial pellet feed and water were allowed* ad libitum*. All animal experiments in this study were performed according to the guidelines of Kangwon National University IACUC (KIACUC-09-0144), and the study was approved by the Ethics Committee of the Kangwon National University.

### 2.4. Scopolamine and Sample Administration

The mice were divided into seven groups (*n* = 7): a control group (the saline treated group), a scopolamine (1 mg/kg) alone treated group, a donepezil (1 mg/kg) treated group as positive control (scopolamine + donepezil), and four concentrations (3, 10, 30, and 100 mg/kg) of ENS treated groups (scopolamine + ENS). The mice were administered donepezil and ENS orally 90 min before subcutaneous treatment with scopolamine. Donepezil is an AChE inhibitor and is widely used in the treatment of AD. Scopolamine was subcutaneously administered 30 min before the water maze test, daily, on four consecutive test days (14:00–18:00 each day). In the passive avoidance test, the mice were administered orally 120 min prior to the start of the training trial. After 90 min, amnesia was induced by scopolamine (1 mg/kg body weight) given subcutaneously.

### 2.5. Morris Water Maze Test

We used the Morris water maze test to evaluate the memory-improving effect of ENS on scopolamine-induced memory deficits in mice. Scopolamine induces memory deficits through acting as a muscarinic acetylcholine receptors antagonist. The test was performed according to the method described previously [15, 16]. A large circular pool (diameter: 90 cm, height: 40 cm) was filled with water (20 ± 1°C) to a depth of 30 cm and divided into four equal quadrants. A white plastic platform was submerged 1 cm below the surface of the water in one quadrant of the pool. A camera linked to a Smart (v.2.5.21) video-tracking system was used to monitor and analyze the swimming activity of the mice. Trial sessions were given to mice each day for four consecutive days. Location of the platform was unchanged but a different start point was used during the test period. Escape latency, or the time taken to locate the platform, was recorded over a 120 s time period. In the probe trial, the platform was removed 24 h after the final test day, and mice were given 60 s to swim without the platform. Swimming time in the quadrant where the platform had previously been placed was recorded. This allowed us to determine the memory function of the mice and the effect of scopolamine on escape latency and time spent in the quadrant that had contained the platform.

### 2.6. Passive Avoidance Test

The passive avoidance test is a useful behavioral study for measuring learning and memory, based on associative emotional learning, by demonstrating an adaptive response to a stressful experience. The apparatus (Gemini system, San Francisco, USA) used for the passive avoidance test consisted of one light and one dark compartment separated by a guillotine with an electrifiable grid floor. On the first day of testing, a training trial was performed. The mouse was placed in the light compartment, and after 40 s the guillotine door was opened. When the mouse moved into the dark compartment, the door closed automatically and an electric foot-shock (0.1 mA/10 g body weight) was delivered through the grid floor for 2 s. The time taken to enter the dark compartment was recorded for each trial. Any mouse that stayed up to 180 s in the light compartment was excluded from the experiment. Twenty hours after the training trial, a test trial was performed and the latency for entering the dark compartment was recorded.

### 2.7. Determination of Acetylcholinesterase Activity in Brain

Acetylcholinesterase (AChE) activity was determined using a spectrophotometric method based on an Ellman method [17]. Mice of each group were euthanized after behavioral test, and brain was rapidly removed. Hippocampus was dissected out from the brain, homogenized containing phosphate buffer (pH 8.0), and, then, centrifuged at 13,000 ×g for 15 min at 4°C. The supernatant was mixed with 470 *μ*L phosphate buffer (pH 8.0), 280 *μ*L of acetylcholine iodide, and 167 *μ*L of 5.5′-dithiobis(2-nitrobenzoic acid) (DTNB). The mixture was incubated at 37°C for 5 min. AChE activity was measured at 412 nm and was expressed in U/mg protein.

### 2.8. Cell Viability

Cell viability was determined by MTT assay as described by a previous method [16]. Glutamate is an excitatory neurotransmitter that plays an important role in brain function, including memory and learning, in the central nervous system (CNS). High concentrations of glutamate are involved in cell death through the glutamate agonist, N-methyl-D-aspartate (NMDA). HT22 is a neuronal cell line derived from the mouse hippocampus and used widely to study mechanisms related to glutamate-induced oxidative stress and cell death [18]. HT22 cells were obtained from the Seoul National University, Korea, and cultured in DMEM supplemented 10% FBS and 1% penicillin-streptomycin. HT22 cells (6.7 × 10^4^/well) were seeded in 48-well plates and incubated for 24 h at 37°C at 5% CO_2_. After 10 and 100 *μ*g/mL of ENS and trolox (50 *μ*g/mL, positive control) treatments, 2 mM glutamate was added and incubated for 24 h. Trolox is an antioxidant that reduces oxidative stress in cells such as vitamin E. After incubation, the cells were treated with the MTT (1 mg/mL) solution for 3 h. Dimethyl sulfoxide (DMSO) was then added to each well and the optical density of the produced formazan by viable cells was measured at 570 nm using an ELISA reader.

### 2.9. ROS Level Measurement

ROS formation induces neuronal cell death by oxidative stress. Glutamate is involved in ROS production through NMDA receptor activation and intercellular Ca^2+^ accumulation. We evaluated ROS production using the dye DCF-DA (2′7′-dichlorofluorescein diacetate) in HT22 cells. Three different concentrations of ENS sample were treated with 2 mM glutamate for 8 h in the seeding cells. Subsequently, 10 *μ*M DCF-DA was added to the cells and then incubated at 37°C for 30 min. After incubation, the DMEM medium was removed and washed twice with PBS (0.01 M, pH 7.4) and extracted with 1% triton X-100 in PBS (0.01 M, pH 7.4) for 10 min at 37°C. Fluorescence was measured at an excitation wavelength of 490 nm and emission wavelength of 525 nm for detection of ROS formation.

### 2.10. Calcium (Ca^2+^) Measurement

High concentrations of glutamate lead to intercellular Ca^2+^ accumulation by activation of NMDA receptors. Increased Ca^2+^ has been implicated in neuronal cell death through depolarization of the mitochondrial membrane. Cytosolic Ca^2+^ concentration was measured using the Fura-2AM in HT22 cells. HT22 cells were cultured at 37°C at 5% CO_2_. 10 and 100 *μ*g/mL of ENS sample, 2 *μ*M Fura-AM, and glutamate were treated in HT22 cells. After 20 min, cells were washed with HEPES buffer saline and incubated for 1 h. Fluorescence was monitored at an excitation wavelength of 380 nm with fixed emission at 510 nm.

### 2.11. DPPH Radical Scavenging Assay

The DPPH (1,1-diphenyl-2-picrylhydrazyl) radical scavenging activity was conducted to evaluate antioxidant activity. Various concentrations of samples were added to 150 *μ*L of 0.4 mM DPPH methanol solution in 96-well plates. Absorbance of DPPH solution was measured using an ELISA reader at 517 nm. DPPH radical scavenging activity was expressed and calculated as %  inhibition = (1 − A_*s*_/A_*o*_) × 100, where “A_*s*_” is sample absorbance and “A_*o*_” is only DPPH solution absorbance.

### 2.12. Hydrogen Peroxide (H_2_O_2_) Scavenging Assay

DPPH radical and H_2_O_2_ scavenging activity were investigated to clarify the antioxidant properties of ENS. The H_2_O_2_ scavenging activity of ENS was determined according to the modified method of Ruch [19]. Different concentrations of ENS were added to the 10 mM H_2_O_2_ in 100 *μ*L of phosphate buffer (pH 0.5, 0.1 M). After incubation at 35°C for 5 min, 1.25 mM ABTS (2,2-azinobis(3-ethylbenzothiazoline)-6-sulfonic acid) and 1 unit/mL peroxide were added and again incubated at 35°C for 10 min. Absorbance of hydrogen peroxide at 230 nm was determined and calculated as %  inhibition = (1 − A_*s*_/A_*o*_) × 100, where A_*s*_ is sample absorbance and A_*o*_ is only H_2_O_2_ solution absorbance.

### 2.13. Statistics

The results of the* in vivo* and* in vitro* tests were expressed as means ± S.E.M. Data from the probe trial of the Morris water maze test, the passive avoidance test, MTT assay, and ROS and Ca^2+^ measurement were analyzed using a one-way analysis of variance (ANOVA) followed by a Tukey's post hoc test. Escape latency in the Morris water maze test was analyzed using a two-way ANOVA followed by a Newman-Keuls test. Statistical significance was set at *P* < 0.05, 0.01, and 0.001.

## 3. Results

### 3.1. Effect of ENS on Scopolamine-Induced Memory Impairment in the Morris Water Maze Test

**Figure 1:**
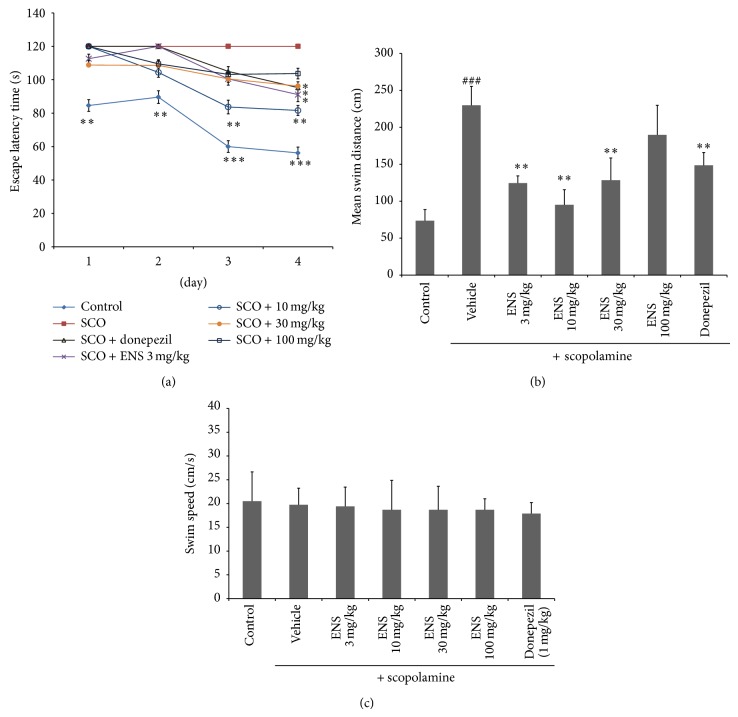
(a) Effect of ENS (3, 10, 30, and 100 mg/kg) on escape latency (s) for 4-day trials of the Morris water maze test in scopolamine-induced memory impairment. Donepezil group (positive control; 1 mg/kg body weight, P.O.) and ENS (3, 10, 30, and 100 mg/kg body weight, P.O.) were treated 90 min before scopolamine administration. The test trial was performed 30 min after scopolamine treatment. Escape latency (s), the time taken to locate the platform, was recorded during the test trial. Results are expressed as the mean escape latency of each group on the test day. Data represent means ± S.E.M. (*n* = 7) (^*^ *P* < 0.05, ^**^ *P* < 0.01, and ^***^ *P* < 0.001 versus the scopolamine group). Effect of ENS (3, 10, 30, and 100 mg/kg) on (b) mean swim distance on day 4 and (c) swim speed during the test trial. Data represent means ± S.E.M. (*n* = 7) (^###^ *P* < 0.001 versus the control group; ^**^ *P* < 0.01 versus the scopolamine group). Control: control group, Sco: scopolamine only treated group, Sco + donepezil: scopolamine and donepezil treated group, and Sco + ENS: scopolamine and ENS treated group.

**Figure 2:**
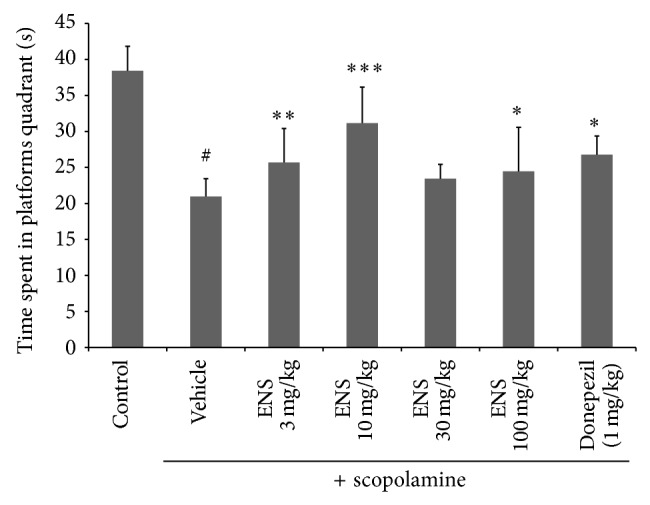
Effect of ENS (3, 10, 30, and 100 mg/kg) for the probe trial in the Morris water maze test. Data represent means ± S.E.M. (*n* = 7) (^#^ *P* < 0.05 versus the control group; ^*^ *P* < 0.05, ^**^ *P* < 0.01, and ^***^ *P* < 0.001 versus the scopolamine group).

We evaluated the escape latency and swimming distance of mice to find the platform (Figure 1(a)) (*n* = 7). A two-way ANOVA revealed significant main effects for trial days (*F*(3,196) = 1.60, *P* < 0.001) and treatment groups (*F*(6,196) = 2.06, *P* < 0.001), but there is no interaction between trial days and treatment groups (*F*(18,196) = 1.29, *P* > 0.05). The control group showed a decrease in escape latency across the four trial days, whereas the scopolamine administration group showed similar escape latencies across the four training days. This relative increase in escape latency when compared to the control group indicated a spatial memory deficit. We found that ENS significantly reduced the escape latency time of mice compared with the scopolamine group. The results showed that, compared with the scopolamine group, ENS significantly decreased the swim distance to platform (Figure 1(b)). Swim speed during the test trial and total swim distance on day 4 were evaluated using a swim path analysis. The swimming speed of mice was not significantly different between the groups (Figure 1(c)). In the probe trial, scopolamine significantly decreased swimming time in the target quadrant compared to the control group, whereas ENS administration significantly increased swimming time in the target quadrant (Figure 2) compared to the control group. Of the four ENS doses, 3 and 10 mg/kg of ENS reversed scopolamine-induced memory deficits compared to the other doses (30 and 100 mg/kg).

### 3.2. Effect of ENS on Scopolamine-Induced Memory Impairment in the Passive Avoidance Test

**Figure 3:**
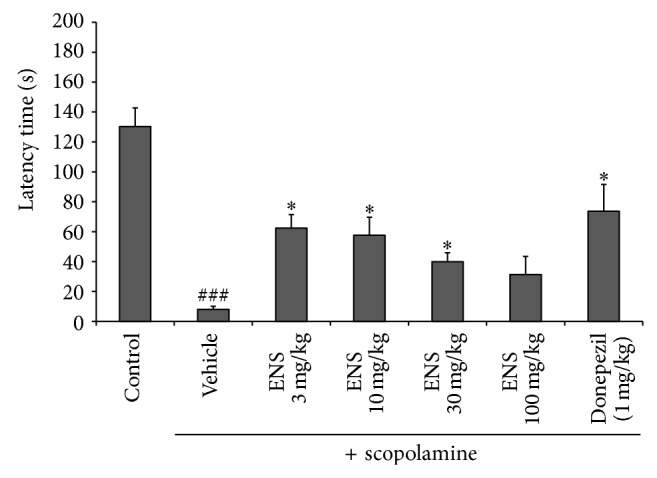
Effect of ENS (3, 10, 30, and 100 mg/kg) in scopolamine-induced memory impairment for the passive avoidance test. Mice were orally administered with ENS (3, 10, 30, and 100 mg/kg body weight, P.O.) and donepezil group (positive control; 1 mg/kg body weight, P.O.) 90 min before scopolamine (1 mg/kg body weight) subcutaneous administration. After 30 min a training trial was performed. Twenty hours after the training trial, a test trial was performed and the latency was measured. Data represent means ± S.E.M. (*n* = 7) (^###^ *P* < 0.001 versus the control group; ^*^ *P* < 0.05 versus the scopolamine group).

Latency was not different between any of the groups during the training trial on the first day. For the test trial, the latency of the scopolamine treated group for entering the dark compartment was significantly shorter than the control group, indicating a memory impairment (Figure 3) (*n* = 7). Treatment with ENS at 3 and 10 mg/kg doses significantly increased latency compared to scopolamine treated group (*F*(2,21) = 14.93, *P* < 0.05). This indicated that ENS improved memory relative to scopolamine-induced memory impairment. Treatment with donepezil, the positive control, also significantly increased latency (*F*(1,14) = 18.77, *P* < 0.05).

### 3.3. Effect of ENS on AChE Activity

**Figure 4:**
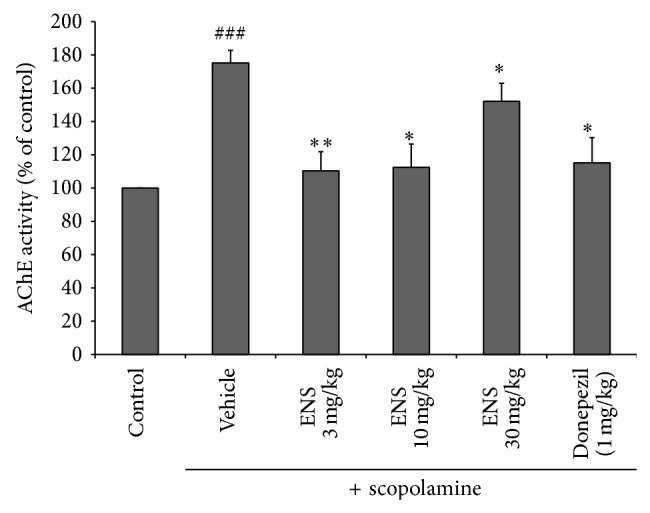
Effect of ENS on AChE activity in scopolamine-induced memory deficit mice hippocampus. Data represent means ± S.E.M. (*n* = 7) (^###^ *P* < 0.001 versus the control group; ^*^ *P* < 0.05 and ^**^ *P* < 0.01 versus the scopolamine group).

To evaluate the effect of AChE inhibition of ENS, AChE activity assay was conducted in hippocampus. AChE activity in hippocampus was significantly increased by scopolamine treatment significantly increased as compared to control group and this AChE activity was significantly inhibited by 37.05% and 35.83% at a dose of 3 and 10 mg/kg of ENS, respectively (*n* = 3) (Figure 4). Increased AChE by scopolamine was also attenuated by donepezil (positive control). Results showed cognitive-enhancing effect of ENS was related to inhibition of AchE in hippocampus.

### 3.4. Effect of ENS on Glutamate-Induced Cell Death in HT22 Cells

**Figure 5:**
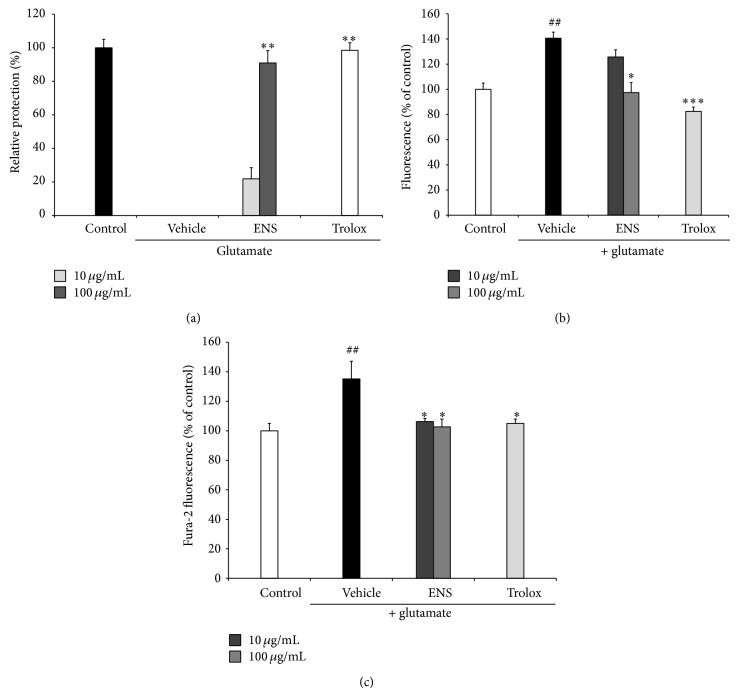
(a) The neuroprotective effect of ENS (10 and 100 *μ*g/mL) on glutamate-induced cell death in HT22 cells. After treatment with the ENS (10 and 100 *μ*g/mL), trolox (50 *μ*g/mL, positive control), and glutamate (2 mM), the optical density of the cell viability was measured at 570 nm and expressed as a relative protection to control cells. (b) Effect of ENS (10 and 100 *μ*g/mL) on ROS production. After treatment with the ENS (10 and 100 *μ*g/mL), trolox (50 *μ*g/mL, positive control), and glutamate (2 mM), cells were incubated with 10 *μ*M DCF-DA. ROS formation was measured at an excitation wavelength of 490 nm and emission wavelength of 525 nm. (c) Effect of ENS (10 and 100 *μ*g/mL) on Ca^2+^ influx in HT22 cells. Cells were treated with ENS (1, 10, and 100 *μ*g/mL) with 2 *μ*M Fura-AM. After 20 min, fluorescence was measured at an excitation wavelength of 380 nm with fixed emission at 510 nm. Data represent means ± S.E.M. of three independent experiments. (^##^ *P* < 0.01 versus the control group; ^*^ *P* < 0.05, ^**^ *P* < 0.01, and ^***^ *P* < 0.001 versus the glutamate treated group).

We investigated the neuroprotective effect of ENS on glutamate-induced cell death in HT22 cells by MTT assay (Figure 5(a)). In glutamate treated cells, the viability of cells was decreased. In contrast, the viability of cells treated with ENS was significantly increased compared to glutamate treated cells. 100 *μ*g/mL of ENS showed 90.84 ± 6.17% cell viability against glutamate-induced cell death (*n* = 3). Trolox, used as a positive control, also significantly reduced cell death by glutamate.

### 3.5. Effect of ENS on ROS Production

Glutamate treated cells increased fluorescence compared to the control group. ENS pretreated cells inhibited ROS overproduction in a dose-dependent manner, with 100 *μ*g/mL of ENS significantly decreasing ROS production to 97.41 ± 7.97%(*F*(1,4) = 11.44, *P* < 0.05) (*n* = 3) (Figure 5(b)). These results indicated that ENS protected HT22 cells against glutamate-induced cell death by inhibiting ROS production.

### 3.6. Effect of ENS on Ca^2+^ Accumulation

As shown in Figure 5(c), glutamate treated cells (135.21 ± 11.96%) increased Ca^2+^ concentration compared to control cells. Doses of 10 and 100 *μ*g/mL of ENS significantly reduced Ca^2+^ concentration induced by glutamate to 106.30 ± 2.13% and 102.66 ± 5.30%, respectively (*F*(1,4) = 18.57, *P* < 0.05) (*n* = 3).

### 3.7. Antioxidant Activity of ENS on DPPH Radical and H_2_O_2_ Scavenging Assay

**Figure 6:**
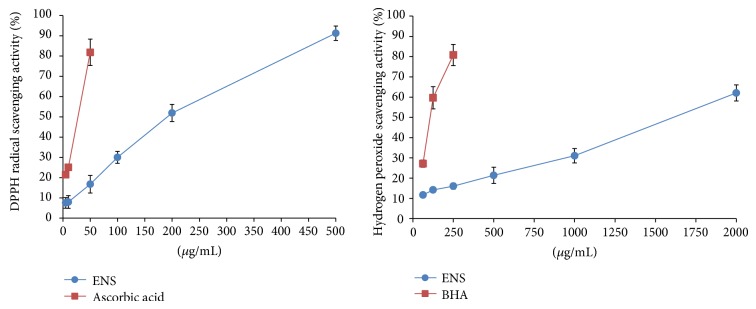
DPPH radical and H_2_O_2_ scavenging activity of ENS. Ascorbic acid and BHA are used as a positive control. Data represent means ± S.E.M. of the three independent experiments.

DPPH radical and H_2_O_2_ scavenging activity of ENS showed IC_50_ (*μ*g/mL) value as 240.51 and 1769.01, respectively (Figure 6). The DPPH radical scavenging activity of ENS was higher than the H_2_O_2_ scavenging activity. Ascorbic acid and BHA were used as a positive control. These results suggested that ENS protected neuronal cells from free radicals such as H_2_O_2_.

## 4. Discussion

The present study demonstrated that ENS ameliorated scopolamine-induced memory impairment in mice and protected HT22 cells against glutamate-induced cell death. In the Morris water maze test, spatial learning and long-term memory function is determined by measuring repeated training trials in which mice have to find a platform, followed by measuring preference for the platform quadrant in a probe trial when the platform is hidden. We found that the administration of ENS significantly decreased escape latency and increased time spent in the target quadrant, compared to the scopolamine treated group. These results indicate that ENS improves spatial learning and memory in a scopolamine-induced dementia mouse model. Moreover, ENS reduced escape latency from day to day, indicating that ENS also improves reference or long-term memory [17].

We found no significant difference between groups for swimming speed during the test trial, demonstrating that ENS and scopolamine did not affect the locomotor activity of mouse and therefore did not influence escape latency. We additionally investigated the swim search pattern of mice on 4 trial day. The swim search pattern is defined in three ways: spatial, nonspatial, and nonsearching. The scopolamine treated group showed a nonsearching pattern and demonstrated thigmotaxic behavior (looping along the side of maze). In contrast, the ENS treated group showed a nonspatial search pattern, which was illustrated by a scanning pattern (searching for the platform location using a spatial bias) [20].

We used a passive avoidance test to assess memory based on associative emotional learning. ENS treatment increased latency time which mice take to move into the dark compartment compared with scopolamine treated group. The data also showed that ENS ameliorated the memory impairment that had been induced by scopolamine. In the Morris water maze test, the effect of ENS (10 mg/kg) was higher than the donepezil treated group. However, the effect of ENS was lower than donepezil treated group in the passive avoidance test. Together, these findings indicate that ENS has a greater effect on spatial learning and memory.

The cholinergic system is important for memory function, and scopolamine induces memory impairment through blockade of the cholinergic system [21, 22]. Previous studies have shown that* Nelumbo nucifera* semen and* Nelumbo nucifera* seedpod inhibit AChE activity [10, 13]. Choline acetyltransferase (CHAT) is an enzyme that is synthesized by acetylcholine, and* Nelumbo nucifera* semen improves memory by inducing CHAT expression. Moreover, neferine, one of the major compounds in ENS, has an antiamnesic effect due to AChE inhibition. Further, we found that ENS inhibited AChE activity in the hippocampus. These findings suggest that the possible mechanism underlying the memory improving effects of ENS, as demonstrated in the Morris water maze and passive avoidance tests, may be related to the cholinergic neuronal system.

In this study, low concentrations (3 and 10 mg/kg) of ENS showed a more potent memory-enhancing effect than the 30 and 100 mg/kg doses. This means that the cognitive-enhancing effect of ENS was not due to increased concentration of ENS extract. Importantly, this finding replicates data reported in previous studies [23, 24]. These results may be related to the sedative effect of ENS and neferine, a major compound in ENS [25]. Previous studies reported that sedation induced memory deficits [26].* Angelica keiskei* and* Mitragyna speciosa* have sedative effect and impaired memory in high concentration [27, 28]. Therefore, ENS can lead to memory deficits in high dosage by sedative effect.

Oxidative stress-induced neuronal cell death is a response observed in neurodegenerative diseases such as AD [29]. To evaluate the effect of ENS against oxidative stress, we used a neuronal cell derived from the mouse hippocampus (HT22) and induced oxidative stress with glutamate treatment. Glutamate excitotoxicity in neuronal cells induces oxidative stress via intercellular Ca^2+^ influx and ROS generation [30]. A high ROS level, including hydroxyl radical (OH^−^), superoxide anion (O_2_^−^), and hydrogen peroxide (H_2_O_2_), has an important role in the pathogenesis of AD [31]. Intercellular Ca^2+^ influx contributes to excessive ROS production via NMDA receptors. ENS showed a neuroprotective effect against glutamate-induced cell death on the HT22 cells. ENS also inhibited ROS generation and Ca^2+^ accumulation in HT22 cells. We found that reduction of ROS production, via inhibition of intercellular Ca^2+^ accumulation, could affect the neuroprotective effect of ENS. ENS also showed antioxidant activity with the DPPH radical and H_2_O_2_ scavenging activity in this study. Taken together, these results suggest that ENS may inhibit oxidative stress-induced cell death through antioxidant activity.

## 5. Conclusion

In conclusion, ENS improved memory in a scopolamine-induced mice model of dementia and protected the neuronal cells from glutamate-induced cell death. The cognitive enhancement and neuroprotective effect of ENS may be related to AChE inhibition, reduction of ROS production, intercellular Ca^2+^ accumulation, and antioxidative activity. Future studies are needed to demonstrate the mechanisms that underpin the positive effects of ENS.
